# Supplementary material for: Crossing chasms: A PLS approach to EU public sector performance assessment
Source: PLoS One. 2025 Jun 25;20(6):e0325994. doi: 10.1371/journal.pone.0325994 (PMC12193962; doi:10.1371/journal.pone.0325994)
Supplement: Attachment — Submitted filename: Response to Reviewers_final.docx [file pone.0325994.s003.docx]

In the attention of:

Emily Chenette

Editor-in-Chief, Journal PLOS ONE

November 8^th^, 2024

Dear Editor-in-Chief,

Dear Anonymous Reviewers,

We express our gratitude for your reconsideration of the manuscript titled “*Bridging Gaps in EU Public Sector Performance Assessment: An Analytical Approach Based on PLS Perspective*” with manuscript ID *PONE-D-24-38083*.

We would like to extend our deepest gratitude to you for your thorough review of our manuscript and for allowing us to revise and enhance the quality of the final version. We are greatly encouraged by your thoughtful feedback, and we are revising our paper with the utmost care and dedication.

In response to the valuable comments provided, we have carefully addressed each point to ensure the overall improvement of the manuscript. Below are our detailed responses to each observation and the corresponding revisions made. Our manuscript has been revised considering the comments from Reviewers 1 and 2, and all changes in the revised manuscript were introduced in *red*. Additionally, we have made several other revisions to enhance the quality of the paper, including improving the grammar and spelling throughout the manuscript.

We believe that these changes to the manuscript will facilitate the decision to publish this study in the *Journal PLOS ONE*. We have made a substantial effort to consider the essential suggestions proposed by the reviewers and remain open to consideration of any further comments on our answers.

Sincerely,

On behalf of the authors,

Assistant Professor, PhD Alexandra-Mădălina Țăran

**JOURNAL REQUIREMENTS**

Manuscript ID: PONE-D-24-38083

Journal: PLOS ONE

Article title: Bridging Gaps in EU Public Sector Performance Assessment: An Analytical Approach Based on PLS Perspective

**Point 1:** Please ensure that your manuscript meets PLOS ONE's style requirements, including those for file naming. The PLOS ONE style templates can be found at

https://journals.plos.org/plosone/s/file?id=wjVg/PLOSOne_formatting_sample_main_body.pdf and

https://journals.plos.org/plosone/s/file?id=ba62/PLOSOne_formatting_sample_title_authors_affiliations.pdf

**Response for Point 1:** Thank you for your insightful comments regarding PLOS ONE’s style requirements, including those for file naming. We appreciate your feedback and have carefully checked the style templates and file naming.

**Point 2:** Please note that PLOS ONE has specific guidelines on code sharing for submissions in which author-generated code underpins the findings in the manuscript. In these cases, we expect all author-generated code to be made available without restrictions upon publication of the work. Please review our guidelines at https://journals.plos.org/plosone/s/materials-and-software-sharing#loc-sharing-code and ensure that your code is shared in a way that follows best practice and facilitates reproducibility and reuse.

**Response for Point 2:** Thank you for clarifying the guidelines on code sharing. We confirm that all author-generated code underlying our findings will be made available without restrictions upon publication. To ensure adherence to PLOS ONE's best practices and facilitate reproducibility and reuse, we will provide clear documentation for the code, including any dependencies or software requirements. The code will be accessible via a stable, public repository, and we will include the relevant URLs or DOIs in our submission. We are committed to supporting transparency and reproducibility and will ensure that our code is shared in full compliance with PLOS ONE’s standards.

**Point 3:** We note that your Data Availability Statement is currently as follows: All relevant data are within the manuscript and its Supporting Information files.

Please confirm at this time whether or not your submission contains all raw data required to replicate the results of your study. Authors must share the “minimal data set” for their submission. PLOS defines the minimal data set to consist of the data required to replicate all study findings reported in the article, as well as related metadata and methods (https://journals.plos.org/plosone/s/data-availability#loc-minimal-data-set-definition).

For example, authors should submit the following data:

- The values behind the means, standard deviations and other measures reported;

- The values used to build graphs;

- The points extracted from images for analysis.

Authors do not need to submit their entire data set if only a portion of the data was used in the reported study.

If your submission does not contain these data, please either upload them as Supporting Information files or deposit them to a stable, public repository and provide us with the relevant URLs, DOIs, or accession numbers. For a list of recommended repositories, please see https://journals.plos.org/plosone/s/recommended-repositories.

If there are ethical or legal restrictions on sharing a de-identified data set, please explain them in detail (e.g., data contain potentially sensitive information, data are owned by a third-party organization, etc.) and who has imposed them (e.g., an ethics committee). Please also provide contact information for a data access committee, ethics committee, or other institutional body to which data requests may be sent. If data are owned by a third party, please indicate how others may request data access.

**Response for Point 3:** Thank you for your detailed guidelines on the Data Availability Statement. We confirm that our submission contains all raw data necessary to replicate the results of our study, adhering to PLOS's definition of the minimal data set. Specifically:

- The values underlying all reported means, standard deviations, and other relevant measures are included in the Supporting Information files.
- The data used to construct all graphs and figures are available for review.
- Points extracted from images for analysis, where applicable, are also included.

If additional information is required, we are open to uploading further Supporting Information files or depositing data in a recommended repository and providing the relevant access details. There are no ethical or legal restrictions on sharing de-identified data for our study, and all data are readily accessible in the submitted materials.

**Point 4:** We note you have included a table to which you do not refer in the text of your manuscript. Please ensure that you refer to Table 2 in your text; if accepted, production will need this reference to link the reader to the Table.

**Response for Point 4:** We apologise for any inconvenience caused by the omitted table that we do not refer to in our manuscript's text. We appreciate your feedback, and in response, we have revised the manuscript and referred to Table 2 in our text:

“As the groundwork for the credibility initiate process only to the Web of Science database and understanding that relevant studies on public sector performance could be omitted, we continue our approach through content analysis, gathering all the identified relevant scientific documents in Table 2.”

**Point 5:** Please review your reference list to ensure that it is complete and correct. If you have cited papers that have been retracted, please include the rationale for doing so in the manuscript text, or remove these references and replace them with relevant current references. Any changes to the reference list should be mentioned in the rebuttal letter that accompanies your revised manuscript. If you need to cite a retracted article, indicate the article’s retracted status in the References list and include a citation and full reference for the retraction notice.

**Response to Point 5:** We thank you for your comments and apologize for the inconvenience. Your recommendations helped us review our reference list to ensure that it is complete and correct. Due to adding a new section in the manuscript, namely the “Discussion” section, the list of references underwent a series of changes, including renumbering the order of references. We confirm that we have not retracted papers from the manuscript. Moreover, while revising our submission, we upload our figure files to the Preflight Analysis and Conversion Engine (PACE) digital diagnostic tool, https://pacev2.apexcovantage.com/. PACE to ensure that figures meet PLOS requirements.

**COMMENTS FROM THE EXTERNAL PEER REVIEWERS**

Manuscript ID: PONE-D-24-38083

Journal: PLOS ONE

Article title: Bridging Gaps in EU Public Sector Performance Assessment: An Analytical Approach Based on PLS Perspective

**Reviewer: 1**

**Please find below specific answers to each comment/observation of Reviewer 1:**

**Point one:** “This paper addresses an interesting topic related to the EU public sector performance assessment. I acknowledge the amount of work invested in preparing the manuscript and consider it significantly contributes to the scientific literature. I would only suggest to the author(s) to enhance the policy implications of own findings and limitations / future research directions, both could be augmented.”

**Response for Point 1:**

We thank you for your comments and apologize for the inconvenience. Your recommendations helped us enhance the policy implications of our findings, alongside the main limitations and future research directions of our research. Considering your valuable feedback, we reported within the manuscript accordingly (coloured in red) as follows:

“In addition, the implications of our results highlight the importance of a public sector performance index, both in terms of findings and limitations. Our main findings also sustain some critical paths of significance for measuring public sector performance. Nevertheless, through the existence of a public sector performance index, decision-makers have to be more responsible and communicative with citizens and stakeholders, ensuring that both parties use the resources efficiently. Moreover, the measurement of public sector performance identifies and highlights the areas in which government improvements are needed, thus guiding public policy adjustments and improving the delivery of quality public services to spur customer satisfaction. On the same lines, our main findings emphasise that having an index that measures the public sector performance facilitates the efficient allocation of resources, directing funding and efforts to programs that exert positive impact and multiple benefits within society. Moreover, transparency, public trust and involvement in government processes are stimulated. The public sector performance assessment can be considered a tool for operating decisions and improving public sector management practices, thus allowing comparisons between different sectors or regions and highlighting the exchange and implementation of best practices.

A distinct focus of our study is dedicated to identifying additional relevant indicators that can be included and measured in our analysis, as well as establishing new measurement procedures and criteria to pave the way for future research directions. This initiative is driven by the recognition that the current framework for evaluating public sector performance may benefit from a broader set of indicators that reflect the complexities of modern governance, particularly in relation to emerging issues such as environmental sustainability and climate change. We aim to continuously update our data by adapting our research framework to include new indicators and dimensions as they emerge. This dynamic approach allows our study to remain relevant and responsive to evolving societal and environmental challenges, particularly those related to climate change. Furthermore, we also consider developing strategies to combat climate change effects based on our analysis results. Therefore, we intend to expand the measurement of the public sector performance by proposing the creation of an index to measure the performance of the public sector and to have the role of an observatory at the European level, accompanied by permanent updates and unlimited availability. This multifaceted approach underscores our recognition of the critical intersection between public sector performance, digital transformation, and environmental sustainability.

The limitations of our research primarily stem from the challenges associated with data availability and the scope of the indicators chosen for analysis. These limitations can impact the overall accuracy and comprehensiveness of our findings, which are crucial for understanding the performance of the public sector in relation to digital transformation and climate change. Furthermore, in certain conditions, another limitation of our research may embody the relatively small group of pillars since there are multiple societal changes, thus not considering all the relevant components that may influence the measurement of the public sector performance. The lack of comprehensive data on specific indicators can lead to gaps in our understanding of how various dimensions of public sector performance are interrelated. Each pillar—be it digitalisation, governance, environmental impact, or societal factors—plays a crucial role in shaping overall performance. Insufficient data may obscure important relationships and nuances that could inform effective policy decisions. Another limitation arises from the relatively small group of pillars included in our analysis. While we aimed to capture key dimensions of public sector performance, societal changes are multifaceted, and many relevant components may not have been considered. This narrow scope can lead to an incomplete understanding of the factors influencing public sector performance.”

Thank you very much for the Reviewer’s 1 valuable suggestions and recommendations.

**Reviewer: 2**

**Please find below specific answers to each comment/observation of Reviewer 2:**

Point 1: “Title: quite long; try to reduce to one sentence (can be removed : An analytical approach based on PLS perspective)”.

**Response for Point 1:**

Thank you very much for your comment. Based on your recommendation, we have restructured the title to better reflect the focus of our study. The new title we propose is “Crossing Chasms: A PLS Approach to EU Public Sector Performance Assessment”.

**Point 2:** “Abstract: very large; try to introduce with the present results from the article”

**Response for Point 2:**

Thank you for your valuable feedback on the abstract. We appreciate your suggestion to introduce specific results. We believe that the revised abstract effectively summarises the study’s scope through the chosen methodology and its key findings while maintaining clarity. Accordingly, we concentrate on the present results from the article as follows:

“This paper examines the dynamics of public sector performance across European Union (EU) countries through a comprehensive methodological framework. This study introduces the European Public Sector Performance Index, a novel approach that employs Partial Least Squares (PLS) econometric modelling and cluster analysis to evaluate public sector performance from 2007 to 2021. By assessing performance across governance, social, and economic dimensions, the research captures the multifaceted nature of public sector efficiency in the EU. Our investigation reveals significant determinants of performance, including governance factors like Control of Corruption, Rule of Law, and Government Effectiveness, as well as economic indicators such as Inflation and social factors like Equity of access to healthcare services and Education Spending. These findings underscore the critical role of transparent governance, economic stability, and equitable social policies in enhancing public sector efficiency. Despite its reliance on secondary data and the PLS method, the study provides new methodological insights and empirical evidence on public sector performance, contributing to the literature with a holistic analysis that integrates digitalisation and well-being. This study’s holistic approach offers actionable insights for policymakers and stakeholders, emphasising the need for robust governance and equitable policies to improve public sector performance across the EU. The omission of certain societal components—such as economic conditions, demographic changes, or cultural factors—may result in a skewed representation of how digital transformation and governance interact. These external factors can significantly influence the effectiveness of digital initiatives and the overall performance of public institutions.”

**Point 3:** “Introduction: NOVELTY + results (better explanation); very short, increase to maximum to 2 pages”

**Response for Point 3:**

Thank you very much for your comments. In response to your suggestion, we have revised the introduction to focus more clearly on the novelty of the study and provide a concise overview of the key results. We have also increased its length to align with your recommendation, ensuring it will increase to maximum 2 pages.

The updated introduction now highlights the unique contributions of our research, including the novelty and main results.

More specifically, we added:

- a brief presentation of the main results, in a separate paragraph: “Our research examines the dynamics of public sector performance across European Union (EU) countries, introducing the European Public Sector Performance Index, a novel approach that employs Partial Least Squares (PLS) econometric modelling and cluster analysis. Overall, our results reveal the dynamics of public sector performance across European Union (EU) countries. The research results capture the multifaceted nature of public sector efficiency by assessing performance across governance, social, and economic dimensions. Thus, our main results underscore the importance of robust governance and equitable policies to enhance public sector performance across the EU, providing valuable insights for policymakers. The composite index constructed across the pillars demonstrated the considerable effects of governance factors, respectively the control of corruption, the protection and promotion of human rights, and the effectiveness of the governments, having considerable importance within the model and emphasising that the transparent and accountable governance structures can further sustain the increase of public sector’s overall performance. Notably, the most significant inferences in fostering effective governance come from a significant control of corruption, while the boost of economic health, social welfare, and robust governance can be achieved through equal access to healthcare services, alongside an increase in the level of financing as regards the education sector. Additionally, our results highlighted that the digital economy acquires low importance in the governance pillar due to a lack of regard for the decision-makers in implementing efficient digital initiatives within public governance.”
- The study novelty: “The research presented in this study offers several novel contributions to the existing specialised literature on public sector performance measurement. The uniqueness of our findings stems from both the diversity of indicators used and the innovative methodologies employed, which collectively enhance our understanding of public sector dynamics, particularly within the context of the EU-27 Member States. While existing literature often constructs public sector performance indices that focus on a limited set of relative pillars, our study fills a significant gap by demonstrating how multiple dimensions can be integrated into a cohesive measurement framework. This multidimensional approach allows for a more nuanced understanding of public sector performance, recognising that traditional indicators alone may not fully capture the complexities of contemporary governance challenges. Second, the application of complex empirical methods further distinguishes our research. By utilising composite indicators at the level of each pillar (governance, economic, and social), we are able to assess the cumulative effects of various indicators. This methodological innovation enables us to construct a more comprehensive overall index of public sector performance. Additionally, our use of cluster analysis facilitates the identification of specific groups of countries based on their performance levels, allowing us to pinpoint dimensions that could significantly enhance public sector outcomes. This approach highlights country-specific stances and regional differences within the EU, thus contributing to tailored policy recommendations for improving performance. Third, the application of complex empirical methods further distinguishes our research. By utilising composite indicators at the level of each pillar (governance, economic, and social), we are able to assess the cumulative effects of various indicators. This methodological innovation enables us to construct a more comprehensive overall index of public sector performance. Additionally, our use of cluster analysis facilitates the identification of specific groups of countries based on their performance levels, allowing us to pinpoint dimensions that could significantly enhance public sector outcomes. This approach highlights country-specific stances and regional differences within the EU, thus contributing to tailored policy recommendations for improving performance. The fourth significant novel element of our research is the proposal for a dashboard designed to monitor and continuously update public sector performance measurement. This initiative, referred to as the European Public Sector Performance Index, aims to function as an observatory within the EU-27 Member States. By providing real-time data and insights, the dashboard will facilitate ongoing evaluations of public sector performance, promoting transparency and accountability while enabling stakeholders to make informed decisions based on up-to-date information. Five, another crucial contribution of our study is the detailed examination of public sector performance levels across European countries. Our findings reveal significant national differences that must be addressed to enhance the overall public sector effectiveness. By highlighting these disparities, we underscore the importance of developing targeted strategies to improve performance in specific contexts, thus fostering greater equity in public service delivery across the EU. Despite the study's reliance on secondary data and the Partial Least Squares (PLS) method, it provides valuable methodological insights and empirical evidence concerning public sector performance. By integrating digitalisation and well-being into the analysis, we contribute a holistic perspective that enriches the existing literature and encourages future research to adopt similar comprehensive approaches.”

**Point 4:** “Methodology: why the authors use only these indicators into the model and only these countries? Present some theoretical explanations for these indicators”

**Response for Point 4:**

Thank you for your insightful comments regarding our methodology section. We appreciate your feedback on the selection of indicators and the focus on specific countries.

In response to your query about the choice of indicators and countries, we have provided detailed theoretical explanations for these decisions in the revised manuscript. Their relevance drove the selection of indicators to capture the public sector’s performance better.

In the revised manuscript, we have included detailed theoretical justifications for these choices to clarify their relevance and address how these indicators and the selected timeframe contribute to a comprehensive assessment of public sector performance. The details added to the Methodology section are the following:

“The chosen indicators have attempted to capture various aspects of public sector performance. The reasoning behind this selection was largely: 1) Governance and Institutional Quality Indicators, are essential to understanding how well public institution's function, these relate to questions such as policy implementation and government integrity, law enforcement and anti-corruption work. Institutions which are strong tend to be associated with better public service delivery and good economic performance. 2) Digital and E-Government Indicators to test that digital transformation is a proxy for innovation in public administration. 3) Social Indicators (Human Development Index, Quality of Life, Life expectancy, Education Spending, Equity of access to healthcare.) are public policy outcomes, key elements of public sector performance. 4) Economic Indicators (GDP per capita, Inflation, Unemployment, Government debt) are just results from proper public administration and good policy highlighting how the public sector contributes to economic development. 5) Infrastructure Quality Indicators (quality of roads, ports, air transport) are vital for the development of economic activity and major areas where the public sector invests.

The countries included in this analysis cover the EU member states over a long period (to 2021 from 2007). The reason for focusing on these countries is that the EU has a common institutional and regulatory framework which enhances some degree of comparability. By including countries at various stages of economic and social development within the EU, the analysis may yield a variety of patterns or constellations of performance across regions. It is also an approach that respects lower public sector efficiency in Eastern as compared to Western EU members – which thus might give valuable policy insights. This combination of indicators and countries provides a comprehensive picture of public sector performance across the EU. It covers governance, social outcomes, digitalization, economic aspects and infrastructure development -all of which are crucial for evaluating and comparing public sector performance in a fast-moving context.”

**Point 5:** “Explain the construction of the model used for analysis”

**Response for Point 5:**

Please find below the response to the reviewer comment:

“The means of analysis for measuring public sector performance in the European Union consists of two stages: the first is applying Partial Least Squares (PLS) regression to its indicators and second is clustering analysis. PLS regression comprised two main steps.

In the first step, the PLS method has been applied sequentially for the three public performance pillars: governance, economic and social. In the second step, the PLS method addressed all indicators to identify the main determinants of public sector performance.

An important stage has been data normalization and indicator Adjustment. Thus, for each indicator, the minimum value is translated into 1 to avoid division by zero and thus, the indicator value becomes $value=value- {min}_{indicator}+1$. A similar approach is also used for the expected value using the following formula $expect value=expected value-{min}_{indicator}+1$ where ${min}_{indicator}$ is the minimum of the indicator value for both equations. Then, we calculate the indicator expected value gap using the formula:

$$gap= \frac{value}{expected value}-1$$

The gap (or performance) is multiplied by the indicator sign to define whether the performance is positive or negative. Finally, the weight assigned to an indicator for a given country is obtained by PLS.

**PLS econometric framework**, developed by Wold [37], allows the construction of predictive models in the presence of many correlated independent variables and comprises three main steps: model formulation, weight vector calculation, indicator weights and performance calculation.

The first step is to formulate the problem by denoting X as the matrix of adjusted indicator values (including gaps and sign adjustments) for all countries and Y as the response matrix representing the outcome of interest (e.g., overall performance score, pillar performance score). PLS aims to find latent variables (components) from X that best predict the response Y by maximising the covariance between projections of X and Y onto these components [38]. The following step computes weight vectors w (for X) and c (for Y) such that covariance between $X_{w}$ and $Y_{c}$ is maximised by iteratively extracting actors and deflating X and Y. Then, the component scores t are calculated as $t=Xw$, where w is the weight vector for the first component, and additional components are extracted similarly after deflation. The PLS regression model is then constructed using these component scores to predict Y. The weights of the indicators in the final composite score are derived from the PLS regression coefficients, reflecting the contribution of each indicator to the overall performance measure.

Finally, for each country in each year, the performance is aggregated by pillars using the following formula:

$${Performance}_{pillar}=\frac{\sum_{indicator \in pillar} {weight}_{indicator}*{Performance}_{indicator}}{\sum_{indicator \in pillar} {weight}_{indicator}}$$

The final score is obtained by transforming the pillar performance to a scale of 0 to 100 using the minimum and the maximum performance for the pillar in each year and each country using the formula:

$${Score}_{pillar}=100* \frac{{Performance}_{pillar}-min({Performance}_{pillar})}{\max\left( {Performance}_{pillar} \right)-min({Performance}_{pillar})}$$

The second applied method, the cluster analysis approach was used to categorise countries based on their performance, measured by the three pillars of public sector performance: governance, economics, and socials. Cluster analysis, a mainstay in statistical data analysis, enables the discernment of natural groupings in complex datasets, facilitating a nuanced understanding of public sector performance across European Union member states. Determining the optimal number of clusters is a critical preliminary step in cluster analysis. For this purpose, the elbow method, as explained by Ketchen and Shook [39], is employed. This method involves conducting K-means clustering across a range of potential cluster numbers and calculating the total within-cluster sum of squares (WSS) for each. The “elbow point” characterised by a marked change in the rate of decrease of WSS, suggests the most appropriate cluster number. This technique balances the need for cluster homogeneity against the parsimony of the clustering solution [40].

Building on the insights gained, K-means clustering, as described by MacQueen [41], is implemented to finalise country groupings. This iterative algorithm divides the dataset into K distinct, non-overlapping clusters, minimising the within-cluster sum of squares. This approach aligns with the methodology employed by Steinley [42], ensuring the robustness of the cluster solutions. The algorithm assigns data points to clusters to minimise the squared Euclidean distance to the cluster centroid, a method proven effective in similar studies like those of Hartigan and Wong [43].

The methodology also relies on a robustness analysis built based on the hierarchical clustering executed, following the method delineated by Ward [44]. This agglomerative approach incrementally builds cluster hierarchies, enabling the detailed examination of data structures. A dendrogram is constructed to visually represent the clustering process, offering insights into the natural groupings of countries. The use of Ward’s method, which minimises within-cluster variance, aligns with similar applications in other studies [45-46], thereby ensuring analytical rigour.

The integration of these methodologies enables a comprehensive and empirically robust cluster analysis, providing a deep understanding of public sector performance patterns across the European Union. The combined application of the elbow method, hierarchical clustering, and K-means clustering ensures the study’s findings are well-grounded in established analytical practices, contributing significantly to public sector performance analysis discourse. The insights derived from this analysis are invaluable for policymakers and stakeholders in benchmarking performance and formulating targeted strategies for public sector improvement”.

**Point 6:** “Discussions: at least 2 pages; separate from the conclusions”

**Response for Point 6:**

We appreciate your feedback regarding the structure of the manuscript. In response to your suggestion, we have carefully reviewed and revised the manuscript to address your concerns. We have now split the content into distinct Results and Discussions sections to enhance clarity and provide a more thorough analysis.

The Results section now focuses specifically on presenting the findings from our statistical analyses, both PLS method and cluster analysis. In the first stage, PLS method was applied in a waterfall of two steps. In the first step, the PLS method has been applied sequentially for the three public performance pillars: governance, economic and social. In the second step, the PLS method addressed all indicators to identify the main determinants of public sector performance. In the second stage, the cluster analysis approach was used to categorise countries based on their performance, measured by the three pillars of public sector performance: governance, economics, and socials. This section provides a detailed account of the data and the outcomes of our various analytical methods, supported by figures.

The Discussion section has been expanded to a separate section, as per your recommendation. This section interprets the results, supported by tables, explores their implications, and provides a detailed discussion of the findings in the context of existing literature and theoretical frameworks.

This revised structure of our research article (with section Results – Subsection 1. An innovative approach to measuring the performance of the European public sector through composite indicators, with 1.1. The key drivers of the European public sector considering the governance pillar & 1.2. The key drivers of the European public sector considering the economic pillar & 1.3. The key drivers of the European public sector considering the social pillar & 1.4. The key drivers of the overall European public sector and Subsection 2. Exploration and identification of performance trends and patterns of the European public sector index through cluster analysis, with 2.1. Analysis of the European Public Sector performance through a k-means clustering approach & 2.2. A robustness analysis of the European Public Sector performance based on a hierarchical clustering approach and with section Discussion) improves the readability and comprehensiveness of our manuscript.

Thank you very much for the Reviewer’s 2 valuable suggestions and recommendations.

On behalf of the authors,

Alexandra-Mădălina ȚĂRAN
