# Supplementary material for: Crossing chasms: A PLS approach to EU public sector performance assessment
Source: PLoS One. 2025 Jun 25;20(6):e0325994. doi: 10.1371/journal.pone.0325994 (PMC12193962; doi:10.1371/journal.pone.0325994)
Supplement: Attachment — Submitted filename: Response to Reviewers.docx [file pone.0325994.s004.docx]

In the attention of:

Emily Chenette

Editor-in-Chief, Journal PLOS ONE

6th of March 2025

Dear Editor-in-Chief,

Dear Anonymous Reviewers,

We express our gratitude for your reconsideration of the manuscript titled “*Bridging Gaps in EU Public Sector Performance Assessment: An Analytical Approach Based on PLS Perspective*” with manuscript ID *PONE-D-24-38083*.

We would like to extend our deepest gratitude to you for your thorough review of our manuscript and for allowing us to revise and enhance the quality of the final version. We are greatly encouraged by your thoughtful feedback, and we are revising our paper with the utmost care and dedication.

In response to the valuable comments provided, we have carefully addressed each point to ensure the overall improvement of the manuscript. Below are our detailed responses to each observation and the corresponding revisions made. Our manuscript has been revised considering the comments from Reviewers 1 and 2, and all changes in the revised manuscript were introduced in *red*. Additionally, we have made several other revisions to enhance the quality of the paper, including improving the grammar and spelling throughout the manuscript.

We believe that these changes to the manuscript will facilitate the decision to publish this study in the *Journal PLOS ONE*. We have made a substantial effort to consider the essential suggestions proposed by the reviewers and remain open to consideration of any further comments on our answers.

Sincerely,

On behalf of the authors,

Lecturer, PhD Alexandra-Mădălina Țăran

**JOURNAL REQUIREMENTS**

Manuscript ID: PONE-D-24-38083

Journal: PLOS ONE

Article title: Bridging Gaps in EU Public Sector Performance Assessment: An Analytical Approach Based on PLS Perspective

**Journal Requirements:**

**Point one: “**Please review your reference list to ensure that it is complete and correct. If you have cited papers that have been retracted, please include the rationale for doing so in the manuscript text, or remove these references and replace them with relevant current references. Any changes to the reference list should be mentioned in the rebuttal letter that accompanies your revised manuscript. If you need to cite a retracted article, indicate the article’s retracted status in the References list and also include a citation and full reference for the retraction notice.”

**Response for Point 1:**

We thank you for your comments and feedback. Your recommendations helped us review our reference list to ensure that it is complete and correct. Due to adding a new section in the manuscript, namely the “Discussion” section, the list of references underwent a series of changes, including renumbering the order of references. We confirm that we have not retracted papers from the manuscript. Moreover, while revising our submission, we upload our figure files to the Preflight Analysis and Conversion Engine (PACE) digital diagnostic tool, https://pacev2.apexcovantage.com/. PACE to ensure that figures meet PLOS requirements.

**COMMENTS FROM THE EXTERNAL PEER REVIEWERS**

Manuscript ID: PONE-D-24-38083

Journal: PLOS ONE

Article title: Bridging Gaps in EU Public Sector Performance Assessment: An Analytical Approach Based on PLS Perspective

**Reviewer: 2**

**Please find below specific answers to each comment/observation of Reviewer 2:**

**Point one:** Dear Author (s);

Dear Editor,

The manuscript has been revised for better interpretations according to the suggestions of the reviewer(s) by including the information required.

The author(s) change the interpretations, results, methodology, and conclusions accordingly, and therefore, the paper is much improved now. The author(s) reduces considerably the article, references, and diversifies the articles cited.

I recommend that this article be published in PLOS ONE.

Congratulations!

**Response for Point 1:**

We sincerely appreciate your thoughtful review and constructive feedback. Your suggestions have been invaluable in refining our manuscript, and we are grateful for your recognition of the improvements made.

We are especially thankful for your recommendation for publication and your encouraging words. It has been a pleasure working on these revisions, and we appreciate your time and effort in evaluating our work.

**Reviewer: 3**

**Please find below specific answers to each comment/observation of Reviewer 3:**

**Point 1: “**Congratulations on your acceptance! Your hard work has paid off, and this is a moment to celebrate. Well done!”

**Response for Point 1:**

Thank you so much for your kind words and congratulations! We truly appreciate your encouragement and support throughout this process. It means a lot to us that you acknowledge the hard work we’ve put into this manuscript.

We are excited about this achievement and grateful for your thoughtful review, which has undoubtedly contributed to improving our work.

**Reviewer: 4**

**Please find below specific answers to each comment/observation of Reviewer 4:**

**Point 1: “**In the introductory section: a) Including recent data or statistics illustrating the current situation of public sector performance; these could provide a stronger justification for the necessity of the study and the chosen methodology b) Although the paper already includes several references, adding a critical discussion on the limitations of previous studies or different perspectives in the literature could more clearly emphasize the original contribution of this study.”

**Response for Point 1:**

We thank you for your comments and apologise for the inconvenience. Based on your recommendation, we have restructured the introductory section in order to provide a stronger justification for the necessity of the study and the chosen methodology and added a critical discussion on the limitations of previous studies or different perspectives in the literature to more clearly emphasize the original contribution of our study.

We kindly consider it worth mentioning that, in academic style for scientific papers, the introduction section usually focuses on establishing the context and justifying the importance of the study rather than presenting detailed statistics. In this case, we can’t integrate the suggestion to include recent data or statistics in order not to overwhelm the reader with numbers, but rather consider the reviewer's suggestion and develop the context and challenges. Your recommendations helped us enhance the introductory section, in order to provide a stronger justification for the necessity of the study and the chosen methodology of our research. Considering your valuable feedback, we reported within the manuscript accordingly (coloured in red) as follows:

“Recent global crises, such as the COVID-19 pandemic, have highlighted even more urgency for developing governance structures, as public administrations around the world have been forced to adapt to new challenges in service delivery, transparency and resource allocation. As digital transformation continues to reshape sectors globally, there is growing recognition of the need for public institutions to leverage technological advances to maintain and improve their performance.

Moreover, specialised literature aims to capture key dimensions of public sector performance, but the changes and challenges in society are complex and multiple, being possible that some studies do not include essential components in the construction of a composite indicator that measures public sector performance, thus leading to an incomplete understanding of the factor that influences public sector performance. In the context of modern challenges, Herasymiuk et al. (2024) consider how the digital transformation process and technological innovations affect public governance, considering digital tools and innovation as important factors in improving the efficiency and transparency of the public governance process, the results of the study highlighting that digital innovations affect the quality and delivery of public services. Alongside, Scupola & Zanfei (2016) focused on the co-evolution of public governance and innovation, highlighting that technological and institutional contexts significantly impact the governance of the public sector, with increased attention to the role of public actors and innovation models in the public sector, confirming that the governance of the public sector is strictly linked to the dissemination of policy and more national information locally, while also highlighting the importance of the increasingly high degree of involvement of users, who are becoming increasingly capable of influencing the pace and direction of the innovation process within societies.

Thus, based on the underpinnings, our research paper considers two new domains in the construction of a composite index that measures public sector performance, respectively “Innovation” and “Digitalization”, that are not considered by other authors or existing statistics in constructing a composite index that measures the performance of public sector, nuancing the original contribution of our study. Therefore, compared to the existing literature, our study offers different perspectives on measuring the efficiency and performance of the public sector, highlighting the need to consider public governance as a continuous and complex process that can be approached from various perspectives, based on which a series of effective strategies can be developed regarding the implementation and enforcement of digital technology to lead to improving the quality of public service, interaction with citizens, but also to establishing an efficient system, characterized by transparency and effectiveness. Unlike previous studies that constructed a composite indicator that measures the performance of the public sector, this study used an analytical approach based on the PLS perspective in order to gather evidence. This research paper bridged the gap between the existing theory and practice of EU public sector performance assessment.”

**Point 2: “**The "Materials and Methods" section is well structured and detailed, providing a good description of the empirical methods used in the study. However, a few improvements could be made to complete and clarify the methodological approach: a) Justification of the Selection of Indicators; b) Details on the validation of the PLS model.”

**Response for Point 2:**

Thank you for your insightful comments regarding our “Materials and Methods” and “Methodology” section. We appreciate your feedback on the selection of indicators and the details on the validation of the PLS model.

In response to your query about the choice of indicators, we have provided detailed theoretical explanations for these decisions in the revised manuscript. Their relevance drove the selection of indicators that better capture the public sector’s performance.

a) In the revised manuscript, we have included detailed theoretical justifications for these choices to clarify their relevance and address how these indicators and the selected timeframe contribute to a comprehensive assessment of public sector performance. The details added to the “Data Collection” section are the following:

“The justification for their inclusion is based on theoretical foundations, empirical evidence, and policy relevance. Governance plays a fundamental role in shaping public sector performance, as strong institutions lead to better service delivery, economic growth, and public trust. Indicators such as policy implementation effectiveness, government integrity, regulatory quality, law enforcement, and anti-corruption efforts are included because they directly impact institutional strength and democratic stability. Empirical studies have shown that countries with higher institutional quality tend to have more efficient public administrations and resilient economies, justifying their inclusion in the analysis (Kaufmann et al., 2011; North, 1990). The digital transformation of public administration is increasingly recognized as a driver of innovation and efficiency. Digital governance indicators—such as e-Government Development Index (EGDI), public sector digitalization, and online service accessibility—serve as proxies for technological innovation in public administration. These indicators are essential in evaluating how digitalization enhances transparency, reduces bureaucratic inefficiencies, and improves citizen engagement (Canton, 2021). Given the growing role of digital tools in governance, their inclusion reflects modern public sector challenges and advancements. Public sector performance is inherently linked to social outcomes, which reflect the quality of governance and policy effectiveness. Indicators such as Human Development Index (HDI), Quality of Life, Life Expectancy, Education Expenditure, and Healthcare Access Equity are selected because they measure the direct impact of public policies on citizens' well-being. These variables are widely used in global performance assessments (e.g., Lives, 2022) and ensure that the study captures both quantitative and qualitative aspects of social progress. Economic stability and growth are both outcomes and determinants of effective public sector governance. Key economic indicators—such as GDP per capita, inflation rate, unemployment rate, and government debt levels—are included because they provide insights into the economic impact of public sector decisions. These indicators allow for assessing the efficiency of fiscal policies, economic resilience, and public sector contributions to economic development. Prior research has demonstrated that well-functioning public administrations positively correlate with sustained economic growth and financial stability (Afonso et al., 2010). Public investment in infrastructure is a crucial determinant of economic activity and citizen well-being. Indicators measuring the quality of roads, ports, and air transport infrastructure are incorporated to evaluate the extent to which public sector investments contribute to long-term development (Martínez-González et al., 2021). These indicators help capture disparities between EU regions and assess whether public infrastructure investments align with economic needs and policy goals.”

b) Moreover, we also added details on the validation of the PLS model in the “Methodology” and “Discussion” sections:

“In order to validate the results, the PLS methodology allows the calculation of the variance explained by the dependent variable in the model, which is preferably as close as possible to 100%, but also Q2, a measure specific to the PLS regression that helps to evaluate the model's ability to generalise when encounters new input data by cross-validating the data. Thus, a value of Q2 above 0.5 indicates a good prediction model. Finally, four sets of indicators were obtained for the governance, economic, social pillars and over European public.”

“Table 7 presents the accuracy metrics of the PLS regression models across the governance, economic, and social pillars, as well as for the overall European public sector index. The results indicate high explained variance and strong predictive power (Q²) across all models, confirming the robustness of the analytical framework. Among the individual pillars, the governance model exhibits the highest explained variance (90.14%) and Q² metric (0.86), highlighting the dominant role of governance-related factors (e.g., Control of Corruption, Rule of Law, Government Effectiveness) in determining public sector performance. The economic pillar model (explained variance = 79.03%, Q² = 0.81) demonstrates that macroeconomic stability factors (e.g., Inflation, Digital Infrastructure, Economic Freedom) significantly shape public sector efficiency. Similarly, the social pillar model (explained variace = 70.76%, Q² = 0.75) confirms the importance of social policies (e.g., Equity of Healthcare Access, Education Spending, Life Expectancy) in assessing public sector outcomes. For the overall European public sector index, the model explains 64.56% of variance, with a Q² of 0.72, indicating that while governance, economic, and social factors collectively drive performance, governance remains the strongest predictor. Overall, the validation results confirm that the PLS regression models are robust, reliable, and well-suited for analyzing public sector performance in the European Union.”

**Table 7. Validation of PLS regression models in the four scenarios**

| **Model** | **Explained variance** | **Q^2^** |
| --- | --- | --- |
| Governance pillar | 90.14% | 0.86 |
| Economic pillar | 79.03% | 0.81 |
| Social pillar | 70.76% | 0.75 |
| Overall European public sector | 64.56% | 0.72 |

**Point 3: “**The Results section presents a detailed and well-structured analysis of European public sector performance using composite indicators and PLS models. Aspects that could be improved or added to complete the section are a) Adding a short introduction reminding the purpose and importance of this analysis in the broader context of the study. This would help to anchor the results within the research objectives of the study; b) Why "Control of Corruption" is considered a major determinant compared to other variables and how these specific variables were chosen for the PLS model; c) It would be useful to include more direct comparisons with previous studies, highlighting how the current results contribute to the existing literature and what new insights they offer.”

**Response for Point 3:**

Thank you very much for your comments. In response to your suggestion, a) we have revised the “Results” section to focus more clearly on the purpose and importance of our analysis in the broader context of the study; b) we explained why "Control of Corruption" is considered a major determinant compared to other variables and how these specific variables were chosen for the PLS model; and lastly, c) we include more direct comparisons with previous studies, highlighting how the current results contribute to the existing literature and what new insights they offer.”

More specifically, we added:

- a brief presentation of the purpose and importance of our analysis, in a separate paragraph: “Our research examines the dynamics of public sector performance across European Union (EU) countries, introducing the European Public Sector Performance Index, a novel approach that employs Partial Least Squares (PLS) econometric modelling and cluster analysis. The importance of our analysis is highlighted by the results that reveal the dynamics of public sector performance across European Union (EU) countries. The research results capture the multifaceted nature of public sector efficiency by assessing performance across governance, social, and economic dimensions.”
- explaination why "Control of Corruption" is considered a major determinant compared to other variables and how these specific variables were chosen for the PLS model: “The World Bank often highlights corruption as one of the most important barriers to development. According to the World Bank’s World Governance Indicators (WGI), Control of Corruption measures the extent to which public power is exercised for private benefit and assesses the effectiveness of governments in combating corruption. The World Bank identifies corruption as a key constraint to improving governance because it undermines the credibility and legitimacy of institutions and hinders equitable economic development. This variable was chosen for its theoretical relevance, empirical support, and alignment with the dimensions of governance that influence public administration performance. Moreover, the World Bank’s governance indicators, including Control of Corruption, are widely accepted metrics for assessing, conceptualizing and measuring the quality of governance (Dimitrov, 2014; Bac, 2001; Cárcaba, 2017; Knoll & Zloczysti, 2011; Vătavu et al., 2022).”
- direct comparisons with previous studies, highlighting how the current results contribute to the existing literature and what new insights they offer: We are very grateful for your feedback and fully agree with the suggestion to include more direct comparisons with previous studies. One of the key innovations of our study is the inclusion of two new dimensions – innovation and digitalization – that have not been extensively addressed in previous research on public sector performance. These dimensions allow us to explore how modern technological advances and innovative approaches can enhance the efficiency and effectiveness of public administrations. In addition, we use a novel methodology to construct a composite indicator for public sector performance that incorporates these new dimensions and provides a more nuanced and comprehensive measure of public sector performance. In our revised manuscript, we explicitly highlight how our main results contributes and extends the existing literature, as well as the unique insights our study provides, respectively:

-section “Introduction”, paragraph 6 and 7, lines 118-181: “Our research examines the dynamics of public sector performance across European Union (EU) countries, introducing the European Public Sector Performance Index, a novel approach that employs Partial Least Squares (PLS) econometric modelling and cluster analysis. Overall, our results reveal the dynamics of public sector performance across European Union (EU) countries. The research results capture the multifaceted nature of public sector efficiency by assessing performance across governance, social, and economic dimensions. Thus, our main results underscore the importance of robust governance and equitable policies to enhance public sector performance across the EU, providing valuable insights for policymakers. The composite index constructed across the pillars demonstrated the considerable effects of governance factors, respectively the control of corruption, the protection and promotion of human rights, and the effectiveness of the governments, having considerable importance within the model and emphasising that the transparent and accountable governance structures can further sustain the increase of public sector’s overall performance. Notably, the most significant inferences in fostering effective governance come from a significant control of corruption, while the boost of economic health, social welfare, and robust governance can be achieved through equal access to healthcare services, alongside an increase in the level of financing as regards the education sector. Additionally, our results highlighted that the digital economy acquires low importance in the governance pillar due to a lack of regard for the decision-makers in implementing efficient digital initiatives within public governance.

The research presented in this study offers several novel contributions to the existing specialised literature on public sector performance measurement. The uniqueness of our findings stems from both the diversity of indicators used and the innovative methodologies employed, which collectively enhance our understanding of public sector dynamics, particularly within the context of the EU-27 Member States. While existing literature often constructs public sector performance indices that focus on a limited set of relative pillars, our study fills a significant gap by demonstrating how multiple dimensions can be integrated into a cohesive measurement framework. This multidimensional approach allows for a more nuanced understanding of public sector performance, recognising that traditional indicators alone may not fully capture the complexities of contemporary governance challenges. Second, the application of complex empirical methods further distinguishes our research. By utilising composite indicators at the level of each pillar (governance, economic, and social), we are able to assess the cumulative effects of various indicators. This methodological innovation enables us to construct a more comprehensive overall index of public sector performance. Additionally, our use of cluster analysis facilitates the identification of specific groups of countries based on their performance levels, allowing us to pinpoint dimensions that could significantly enhance public sector outcomes. This approach highlights country-specific stances and regional differences within the EU, thus contributing to tailored policy recommendations for improving performance. Third, the application of complex empirical methods further distinguishes our research. By utilising composite indicators at the level of each pillar (governance, economic, and social), we are able to assess the cumulative effects of various indicators. This methodological innovation enables us to construct a more comprehensive overall index of public sector performance. Additionally, our use of cluster analysis facilitates the identification of specific groups of countries based on their performance levels, allowing us to pinpoint dimensions that could significantly enhance public sector outcomes. This approach highlights country-specific stances and regional differences within the EU, thus contributing to tailored policy recommendations for improving performance. The fourth significant novel element of our research is the proposal for a dashboard designed to monitor and continuously update public sector performance measurement. This initiative, referred to as the European Public Sector Performance Index, aims to function as an observatory within the EU-27 Member States. By providing real-time data and insights, the dashboard will facilitate ongoing evaluations of public sector performance, promoting transparency and accountability while enabling stakeholders to make informed decisions based on up-to-date information. Five, another crucial contribution of our study is the detailed examination of public sector performance levels across European countries. Our findings reveal significant national differences that must be addressed to enhance the overall public sector effectiveness. By highlighting these disparities, we underscore the importance of developing targeted strategies to improve performance in specific contexts, thus fostering greater equity in public service delivery across the EU. Despite the study’s reliance on secondary data and the Partial Least Squares (PLS) method, it provides valuable methodological insights and empirical evidence concerning public sector performance. By integrating digitalisation and well-being into the analysis, we contribute a holistic perspective that enriches the existing literature and encourages future research to adopt similar comprehensive approaches.”

-section “Conclusion”, paragraph 13 and 14, lines 1230-1262: “In addition, the implications of our results highlight the importance of a public sector performance index, both in terms of findings and limitations. Our main findings also sustain some critical paths of significance for measuring public sector performance. Nevertheless, through the existence of a public sector performance index, decision-makers have to be more responsible and communicative with citizens and stakeholders, ensuring that both parties use the resources efficiently. Moreover, the measurement of public sector performance identifies and highlights the areas in which government improvements are needed, thus guiding public policy adjustments and improving the delivery of quality public services to spur customer satisfaction. On the same lines, our main findings emphasise that having an index that measures the public sector performance facilitates the efficient allocation of resources, directing funding and efforts to programs that exert positive impact and multiple benefits within society. Moreover, transparency, public trust and involvement in government processes are stimulated. The public sector performance assessment can be considered a tool for operating decisions and improving public sector management practices, thus allowing comparisons between different sectors or regions and highlighting the exchange and implementation of best practices.

A distinct focus of our study is dedicated to identifying additional relevant indicators that can be included and measured in our analysis, as well as establishing new measurement procedures and criteria to pave the way for future research directions. This initiative is driven by the recognition that the current framework for evaluating public sector performance may benefit from a broader set of indicators that reflect the complexities of modern governance, particularly in relation to emerging issues such as environmental sustainability and climate change. We aim to continuously update our data by adapting our research framework to include new indicators and dimensions as they emerge. This dynamic approach allows our study to remain relevant and responsive to evolving societal and environmental challenges, particularly those related to climate change. Furthermore, we also consider developing strategies to combat climate change effects based on our analysis results. Therefore, we intend to expand the measurement of the public sector performance by proposing the creation of an index to measure the performance of the public sector and to have the role of an observatory at the European level, accompanied by permanent updates and unlimited availability. This multifaceted approach underscores our recognition of the critical intersection between public sector performance, digital transformation, and environmental sustainability.”

**Point 4: “**In the Conclusions Section the findings on digital economy factors are interesting, but it would be useful to explore further why these issues did not have a significant impact in the governance pillar and what this might mean for future research and policy implementation.”

**Response for Point 4:** We thank you for your comments and apologise for the inconvenience. Your recommendations helped us enhance our results and what they might mean for future research and the policy implications of our findings. Considering your valuable feedback, we reported within the manuscript accordingly (coloured in red) as follows:

“Different research studies address detailed analysis of potential factors contributing to the limited impact of digital economy variables on governance [Androniceanu, 2022; Lyu, 2024; Niu, 2022]. This research perspective could include considerations such as the different levels of digital infrastructure and digital literacy in different regions, the complexity of integrating digital tools into governance systems, and the possible mismatch between digital transformation and political and institutional contexts. Nevertheless, Zhao [2015] the fact that in most countries where the digital economy has been integrated, these do not have major and significant effects on governance given that citizens do not actively participate in the digital process, thus there is a low level of electronic participation opportunities, public decision-makers need to focus more on the acceptance of electronic services by citizens and on the highest possible degree of use in public sector governance structures, the main focus needs to remain on correcting the existing digital gaps. Policymakers may, therefore, need to adopt a more nuanced and context-specific approach when implementing digital initiatives to ensure that digital economy factors can have a more tangible and positive effect on governance structures.”

Thank you very much for the Reviewer’s 4 valuable suggestions and recommendations.

**Additional changes:**

In accordance with recent institutional restructuring, we would like to update the departmental affiliation for authors Lobont Oana-Ramona and Taran Alexandra Madalina. The department has undergone a reorganization, and as a result, the previous name "Finance Department" has been revised to "Finance, Business Information Systems and Modelling Department." We kindly request that this updated departmental name be reflected in the manuscript to ensure accuracy and consistency with the current institutional structure.

Thank you for your understanding and for incorporating this change.

On behalf of the authors,

Alexandra-Mădălina ȚĂRAN
